# Studying insulin needs in Type 1 Diabetes by analysing the OpenAPS Data Commons

**DOI:** 10.23889/ijpds.v8i3.2271

**Published:** 2023-09-11

**Authors:** Isabella Degen, Kate Robson Brown, Zahraa S. Abdallah

**Affiliations:** 1 University of Bristol

## Introduction & Background

Type 1 Diabetes (T1D) is a chronic condition where the body produces too little insulin, a hormone required to regulate blood glucose (BG). Finding the correct insulin dose and time remains a complex and as yet unsolved control task. Many factors such as food, exercise, stress, menstrual cycle, etc. change how much insulin is required. Most of these factors remain unobserved and/or underexplored. The NHS national diabetes audit shows that in 2020-21 only 9.8% of people with T1D had a NICE recommended glycated haemoglobin (HbA1c) test result of 48 mmol/mol or less to avoid complications due to diabetes.

## Objectives & Approach

Our research aims to improve the understanding of underexplored factors that drive changes in insulin needs of people with T1D. We use unsupervised time series pattern detection methods such as time series k-means, heatmaps and matrix profile to identify underexplored patterns. These are times when insulin on board (IOB) does not rise with more carbohydrates on board (COB) and times when BG does not fall with more IOB and/or rise with more COB as expected. In the future, we aim to use causal feature selection methods to exclude COB as a causal driver for these patterns and identify other factors as causal drivers.

## Relevance to Digital Footprints

We use a digital footprints dataset of n=187 people who use an open-source automated insulin delivery system and have donated their data via the OpenAPS Data Commons and the OpenHumans.org platform. The data has been collected in real-life conditions and contains system logs, BG sensor data and various user-entered annotations. The richness of the data provides a unique opportunity to study what happens in real-life conditions but also poses challenges for many methods. These include inconsistencies between users, irregular sampling between devices and missing data.

## Results

Our pattern detection methods can successfully identify underexplored patterns in insulin needs that are not directly driven by COB. These include months that require more/less insulin without eating more/less carbohydrates and times of day when blood glucose does not rise with more COB and/or fall with more IOB.

## Conclusions & Implications

While our current methods can identify underexplored patterns in insulin needs, the principal pattern identified remains the well-known pattern of the main meal COB spikes. This is not surprising given the frequency and impact of this pattern. We are working on methods that can identify both frequent and less frequent patterns as well as patterns arising from the irregularity of the sampling. Digital footprints datasets like the OpenAPS Data Commons are promising to help increase understanding of complex conditions such as T1D. More of this type of multi-year data is needed. Especially data that includes multiple different sensor readings to help identify causal drivers behind underexplored patterns of insulin needs. And we need pattern-finding methods that deal well with missing and irregularly sampled data.

